# Draft Genome Sequence of a White Spot Syndrome Virus Isolate Obtained in Ecuador

**DOI:** 10.1128/genomeA.00605-18

**Published:** 2018-06-28

**Authors:** Leda Restrepo, Alejandro Reyes, Leandro Bajaña, Irma Betancourt, Bonny Bayot

**Affiliations:** a Escuela Superior Politécnica del Litoral (ESPOL), Centro Nacional de Acuicultura e Investigaciones Marinas, CENAIM, Guayaquil, Ecuador; b Department of Biological Sciences, Universidad de los Andes, Bogotá, Colombia; c Max Planck Tandem Group in Computational Biology, Universidad de los Andes, Bogotá, Colombia; d Center for Genome Sciences and Systems Biology, Department of Pathology and Immunology, Washington University in Saint Louis, St. Louis, Missouri, USA; e Escuela Superior Politécnica del Litoral (ESPOL), Facultad de Ingeniería Marítima, Ciencias Biológicas, Oceánicas y Recursos Naturales, FIMCBOR, Guayaquil, Ecuador

## Abstract

White spot syndrome virus (WSSV) is the most devastating viral disease affecting cultivated shrimp around the world. Currently, there is no reported genetic information on WSSV affecting Penaeus vannamei in Ecuador. Therefore, we determined the genome of a WSSV isolate from shrimp and compared it with the genomes of isolates from other geographical locations.

## Abstract (precis)

White spot syndrome virus (WSSV) is the most devastating viral disease affecting cultivated shrimp around the world. Currently, there is no reported genetic information on WSSV affecting Penaeus vannamei in Ecuador.

## GENOME ANNOUNCEMENT

Aquaculture is one of the most important economic activities in Ecuador, with Penaeus vannamei shrimp being the second highest nonoil export product, accounting for 95% of the country's total aquaculture production (1). The presence of the pathogen white spot syndrome virus (WSSV) in Ecuador was confirmed in 1999 (2). In subsequent years, the disease became an epidemic, resulting in the virus being labeled as the most devastating pathogen in the aquacultural history of shrimp farming. A monitoring effort was carried out along the Ecuadorian coast to evaluate the spread of the virus. Genotyping of the circulating WSSV types was performed to characterize the strain diversity within the country, leading to the sequencing of the complete genome of the most prevalent strain in this territory.

A whole-genome sequencing (WGS) strategy was performed on an Illumina HiSeq sequencer using DNA extracted from infected shrimp containing strain WSSV-EC-15098. In total, 20,649,280 paired-end 2 × 101-bp reads were obtained, which generated 2.085 Gb of total sequenced bases. The reads were mapped to the reference genomes of WSSV strain CN01 (3) (GenBank accession no. KT995472) and WSSV K-LV1 (4) (GenBank accession no. JX515788) using Bowtie 2 (5). They were then used for *de novo* assembly with SPAdes (6). The initial assembly generated 13 contigs. Reads mapping to the edges of the contigs were extracted and assembled using CAP3 (7), and the resulting contigs were used to close the assembly, obtaining a single contig of 288,997 bp. Open reading frames (ORFs) were predicted using the GeneMarkS software, with default parameters (8). The predicted protein sequences were searched against the GenBank database and the Clusters of Orthologous Groups (COGs) protein database using BLASTp (9).

The draft genome of WSSV-EC-15098 consists of 288,997 bp, with a mean G+C content of 40.92%. A total of 162 coding sequences (CDS) were predicted. The genomic architectures were compared with strains from China (3), Thailand (10), South Korea (4), and Mexico (11) using Mauve (12). A complete synteny among the assembled genomes was observed. Two major deletions were observed in the WSSV-EC-15098 genome. The deletions were verified by paired-end reads located at each side of the deletion and mapping to the reference genome without any read mapping to any part of the deleted segments. The deletions are a 3.24-kb fragment containing the ORFs VP22 and VP25, all annotated as envelope proteins, and a 17.04-kb fragment including the ORF VP35, annotated as a capsid protein. The deletion of proteins related to the envelope is very interesting due to its potential role in modifying the way the virus interacts with the host in terms of recognition of the host cell and by the immune machinery. Additionally, it has been observed that strain WSSC-EC-15098 is widely spread but causes the lowest shrimp mortality rate.

The average nucleotide identity (ANI) between WSSV-EC-15098 and other genomes is between 99.67% and 99.94%, with the WSSV-China genome being the most closely related and WSSV-Korea genome being the most distantly related. The implementation of molecular epidemiology for the control of diseases could help in the development of strategies for the prevention and control of emerging and reemerging aquaculture-related diseases.

### Accession number(s).

This whole-genome shotgun project has been deposited at DDBJ/EMBL/GenBank under the accession no. MH090824.
